# Use of complementary and alternative medicine by mid-age women with back pain: a national cross-sectional survey

**DOI:** 10.1186/1472-6882-12-98

**Published:** 2012-07-18

**Authors:** Alex F Broom, Emma R Kirby, David W Sibbritt, Jon Adams, Kathryn M Refshauge

**Affiliations:** 1 School of Social Science, University of Queensland, St Lucia, QLD, 4072, Australia; 2 School of Nursing, Midwifery and Health, University of Technology Sydney, Ultimo, NSW, 2007, Australia; 3 Faculty of Health, University of Sydney, Lidcome, NSW, 2141, Australia

**Keywords:** Back pain, Complementary medicine, Survey

## Abstract

**Background:**

The use of complementary and alternative medicine (CAM) has increased significantly in Australia over the past decade. Back pain represents a common context for CAM use, with increasing utilisation of a wide range of therapies provided within and outside conventional medical facilities. We examine the relationship between back pain and use of CAM and conventional medicine in a national cohort of mid-aged Australian women.

**Methods:**

Data is taken from a cross-sectional survey (n = 10492) of the mid-aged cohort of the Australian Longitudinal Study on Women’s Health, surveyed in 2007. The main outcome measures were: incidence of back pain the previous 12 months, and frequency of use of conventional or CAM treatments in the previous 12 months.

**Results:**

Back pain was experienced by 77% (n = 8063) of the cohort in the previous twelve month period. The majority of women with back pain only consulted with a conventional care provider (51.3%), 44.2% of women with back pain consulted with both a conventional care provider and a CAM practitioner. Women with more frequent back pain were more likely to consult a CAM practitioner, as well as seek conventional care. The most commonly utilised CAM practitioners were massage therapy (26.5% of those with back pain) and chiropractic (16.1% of those with back pain). Only 1.7% of women with back pain consulted with a CAM practitioner exclusively.

**Conclusions:**

Mid-aged women with back pain utilise a range of conventional and CAM treatments. Consultation with CAM practitioners or self-prescribed CAM was predominantly in addition to, rather than a replacement for, conventional care. It is important that health professionals are aware of potential multiple practitioner usage in the context of back pain and are prepared to discuss such behaviours and practices with their patients.

## Background

The use of complementary and alternative medicine (CAM) [1] has increased significantly in Australia over the past decade and is now widespread amongst the general populations of most developed countries [2,3]. One of the most common areas of CAM use is that of musculoskeletal care and in the context of back pain in particular, studies have illustrated that people use a wide range of therapies within and outside the conventional medical domain [4,5].

### The significance of back pain

The significance of back pain for Australian primary care delivery is evident in the fact that it is the second most common complaint in general practice consultations [6,7] representing a key public health problem [8]. Back pain carries both high direct and indirect costs, including reduced capacity to work and participate in community life. For some back pain sufferers conventional treatments have limited therapeutic affect [8,9] leading often to the use of various ‘alternatives’. Back pain care is delivered primarily by general practitioners (GPs), physiotherapists, chiropractors, osteopaths, acupuncturist, and massage therapists. Yet the actual usage and popularity of such modalities amongst Australians remains relatively unknown.

Studies internationally suggest that back pain sufferers often integrate a range of treatments offered by a range of different health care providers. This utilisation of multiple providers may also present difficulties in negotiating multiple and even conflicting models of care [8]. The limited research available suggests that people with back pain often lack confidence in their providers and the treatments they use [4,10]. There is evidence suggesting confusion amongst GPs regarding the role, appropriateness, benefits and risks of the many different treatments available to treat back pain [11]. Limited exploratory research suggests that many Australian GPs are reluctant about, or reject, the assumptions underlying many treatment options for back pain (i.e. chiropractic and osteopathy) and are therefore resistant to referring beyond physiotherapy [11,12]. GPs are more likely to suggest circumstances when patients request CAM providers such as chiropractic and osteopathy [12]. Despite the challenges of multiple providers in the context of back pain care, we know little about what treatments and practitioners Australians are utilising.

### The role of CAM in back pain care

While the efficacy and use of CAM practices is debated, what is clear is that CAM therapies and therapists are playing an important role in the management and treatment of back pain [7,9]. For example, existing Australian data shows chiropractic, acupuncture and osteopathy are frequently utilised by back pain sufferers [5,7]. But the extent to which CAM practices are utilised in isolation from conventional practices is not well understood. While CAM use for management of back pain may be increasingly widespread there is some evidence that people are unwilling to utilise CAM as an exclusive source of CAM for their back pain [7].

The issues presented by the utilisation of competing or conflicting provider groups should not be underestimated. Studies have consistently revealed the lack of communication between patients and doctors about use of CAM [13-15]. It is often suggested that patients are reluctant to disclose their CAM use to their GP or doctor given the traditionally sceptical view of the medical community toward CAM regarding efficacy and risk [3,13,15]. Ultimately, consulting a range of professional groups often involves exposure to competing or conflicting claims to legitimacy and risk, leaving patients with difficult choices regarding who to consult and what advice to draw on [16].

### Risk and efficacy in CAM for musculoskeletal care

In addition to issues of non-disclosure and disconnections in care provision between CAM and conventional medicine, there are concerns surrounding the potential for some CAM therapies to have adverse effects in the context of back pain [14,17,18]. While the level of CAM integration in back pain care has not been well documented, the simultaneous use of CAM and conventional medicine has historically been concerning for the medical community, given the varying and debated claims for legitimacy of evidence for the safety of some CAM practices. This in turn creates an environment of potential liability for general practitioners when referring to CAM providers if such adverse effects are experienced [19].

Ultimately, we know very little about the range of practitioners and practices people are using in Australia for back pain. We know CAM is widely utilised yet we acknowledge here that the broader efficacy and safety of individual CAM modalities are often ambiguous [17,18]. The extent to which various CAM treatments constitute efficacious practices is also highly contested, and we know that acupuncture, chiropractic, osteopathy, and massage therapy are popular CAM options for women seeking back pain care [5,7], but opinions differ on the validity, efficacy and safety of each of these CAM practices [20,21]. Evidence from controlled trials offers little confirmation of the efficacy of individual CAM treatments in comparison to conventional care [20]. This study provides a national, representative perspective on what practices and practitioners mid-age Australian women with back pain are utilising including quality of life indicators and levels of satisfaction with conventional services.

## Methods

### Sample

This research was conducted as part of the Australian Longitudinal Survey on Women’s Health (ALSWH) which was designed to investigate multiple factors affecting the health and well being of women over a 20-year period. Women in three age groups (“young” 18–23, “mid age” 45–50 and “older” 70–75 years) were randomly selected from the national Medicare database [22]. The focus of this study on women reflects existing evidence of CAM use as more commonly used by women than men [23,24]. Further, the focus on the mid-age cohort is in line with a research interest in the physical and psychological impacts of back pain within the context of “ageing well”, an Australian National Research Priority. The baseline survey, comprising of 14099 women, was conducted in 1996 and the respondents have been shown to be broadly representative of the national population of women in the target age groups [25]. Ethics approval was granted by The University of Queensland^a^ and The University of Newcastle^b^ Human Research Ethics Committees, and written informed consent was obtained from all participants. Analyses for this research are restricted to the 10638 women who completed the most recent survey conducted in 2007.

### Measures of health service use

The women were asked about their frequency of use in the previous twelve months of a GP and/or a specialist doctor. In addition, they were asked if they had consulted with a range of conventional providers (i.e. hospital doctor, physiotherapist) and CAM^c^ practitioners (i.e. chiropractor, massage therapist, acupuncturist, naturopath/herbalist, other CAM practitioner).

### Measure of health status

The Short-Form 36 (SF-36) Quality of Life questionnaire was used to produce a measure of health status and quality of life [26]. Results of the SF-36 were reported in eight subscales [26]. The women were also asked whether a doctor had diagnosed or treated them for any of the chronic medical conditions listed, in the previous three years. The list included: arthritis, diabetes, impaired glucose tolerance, heart disease, hypertension, stroke, low iron, asthma, bronchitis, osteoporosis, anxiety disorder, depression, and cancer.

### Rating of health care providers/services

The women were asked to rate their level of satisfaction with various aspects of conventional health care providers (such as access to a female GP, hours when a GP is available, outcomes of medical care). Each aspect was rated via a 5 point Likert scale, where 1 = excellent and 5 = poor.

### Outcome measure

Women were asked how often they had experienced back pain in the previous twelve months^d^.

### Statistical analyses

Comparisons between CAM user status and conventional care provider consultations and rating of conventional health care providers were made using chi-square tests. Comparisons between the means scores on the SF-36 dimensions and CAM user status were made using analysis of variance (ANOVA) tests. All analyses utilised Bonferroni correction for pairwise comparisons. All analyses were conducted using the statistical program SAS version 9.2.

## Results

A total of 10638 women completed the survey, of which 10492 (98.6%) answered the question regarding how often they had experienced back pain. There were 2044 (19.5%; 95%C.I.: 18.7%-20.2%) women who experienced back pain often, 3731 (35.6%; 95%C.I.: 34.7%-36.5%) women who experienced back pain sometimes, 2268 (21.6%; 95%C.I.: 20.8%-22.4%) women who experienced back pain rarely, and 2449 (23.3%; 95%C.I.: 22.5%-24.2%) women who did not have back pain.

### Consultation patterns amongst women with back pain

Consultation with at least one CAM practitioner was made by 4444 (42.4%; 95%C.I.: 41.4%-43.3%) of women in the previous twelve month period. The women with back pain were more likely to consult with a CAM practitioner (45.9%; 95%C.I.: 45.4%-46.5%) than the women without back pain (31.1%; 95%C.I.: 29.3%-32.9%) and this likelihood increased with frequency of back pain (never = 31.1%, rarely = 37.2%, sometimes = 47.6%, often = 52.5%) (p <0.0001). Women with back pain were also more likely to consult with GPs, specialist doctors, hospital doctors and physiotherapists and this likelihood also increased with frequency of back pain (p <0.0001) (data not shown). Table 1 shows that just over a half of women with back pain only consulted with a conventional care provider (51.3%; 95%C.I.: 50.2%-52.4%), while 44.2% (95%C.I.: 43.6%-44.8%) of women with back pain consulted with both a conventional care provider and a CAM practitioner. This shows that almost half the women with back pain utilise both CAM and conventional practices and practitioners. Yet, despite this, conventional medicine maintains a central position within service provision with only 1.7% (95%C.I.: 1.4%-2.0%) of women with back pain consulting with a CAM practitioner but *not* a conventional care provider. Further, we found that as the frequency of women’s back pain increases, so too does the likelihood that they will consult with *both* a conventional care provider and CAM practitioner (p <0.005).

**Table 1:** Back pain status by consultations with conventional health care providers and CAM practitioners

**Consultations**	**Did not have back pain (n = 2,449)**	**Had back pain – rarely (n = 2,268)**	**Had back pain – sometimes (n = 3,731)**	**Had back pain – often (n = 2,044)**	**Total back pain (n = 8,043)**
	**%**	**%**	**%**	**%**	**%**
**Conventional only**	62.8	58.6	49.4	46.6	51.3
**CAM only**	1.7	2.3	1.8	1.0	1.7
**CAM and conventional**	29.4	35.0	45.9	51.5	44.2
**Neither conventional or CAM**	6.1	4.1	2.9	0.9	2.8

Table 2 shows the utilisation of individual CAM practitioner groups according to back pain status. The most commonly utilised CAM practitioner groups were massage therapy (18.7% without back pain, 26.5% with back pain: 95% CI: 25.7%- 27.4%) followed by chiropractic (7.9% without back pain, 16.1% with back pain: 95% CI: 15.4%-16.8%). The data presented in Table 2 highlight the increased likelihood of consultation with all individual CAM practitioner groups as the frequency of back pain increases. For all individual CAM modalities listed, consultations increased considerably in the context of more frequent back pain. Each of the individual practitioner groups were utilised least by women who had no back pain, and most by women who had back pain “frequently”. These data strongly infer that back pain represents a important reason for consultation with various individual CAM practitioners.

**Table 2:** Back pain status by consultations with individual CAM practitioner groups

**Consultations**	**Did not have back pain (n = 2,449)**	**Had back pain – rarely (n = 2,268)**	**Had back pain – sometimes (n = 3,731)**	**Had back pain – often (n = 2,044)**	**Total back pain (n = 8,043)**
	**%**	**%**	**%**	**%**	**%**
Massage therapist (n = 2767)	18.7	24.3	29.1	33.8	26.5
Chiropractor (n = 1681)	7.9	10.9	21.5	22.0	16.1
Naturopath/herbalist (n = 1038)	9.2	9.4	10.0	12.7	10.0
Acupuncturist (n = 627)	3.9	4.7	6.1	9.9	6.0
Osteopath (n = 422)	2.3	2.9	4.7	6.4	4.1
Other CAM practitioner (n = 735)	5.9	5.9	7.8	8.9	7.1

Table 3 shows consultations with conventional health providers according back pain status and CAM consultation. When considering the frequency of back pain categories separately, there are no statistically significant associations between consultation with a CAM practitioner and consultations with GPs, specialists or hospital doctors (Table 4). However, for all back pain categories, women were more likely to consult with a physiotherapist if they also consult with a CAM practitioner (p <0.005).

**Table 3:** Back pain status and consultations with conventional health care providers by CAM user status (consulted with a CAM practitioner or not)

**Consultations**		**Did not have back pain**		**Had back pain – rarely**		**Had back pain – sometimes**		**Had back pain – often**	
		**CAM non-user**	**CAM user**	**CAM non-user**	**CAM user**	**CAM non-user**	**CAM user**	**CAM non-user**	**CAM user**
		**(n = 1,682)**	**(n = 758)**	**(n = 1,419)**	**(n = 842)**	**(n = 1,950)**	**(n = 1,774)**	**(n = 970)**	**(n = 1,070)**
		**%**	**%**	**%**	**%**	**%**	**%**	**%**	**%**
**GP**^**1**^	0	10	7	7	7	6	5	2	3
	1-2	42	35	40	40	35	33	20	19
	3-4	28	32	30	30	30	31	29	27
	5+	19	26	23	23	29	31	49	51
**Specialist Doctor**^**1**^	0	61	55	59	55	54	50	45	39
	1-2	28	31	29	33	31	34	32	35
	3+	11	14	12	12	15	16	23	26
**Hospital Doctor**	no	87	84	85	84	82	80	73	75
	yes	13	16	15	16	18	20	27	25
**Physiotherapist**^**1 2 3 4**^	no	89	80	86	80	82	73	72	62
	yes	11	20	14	20	18	27	28	38

^1^ statistically significant association for women who did not have back pain (*p <.005*).

^2^ statistically significant association for women who had back pain, rarely (*p <.005*).

^3^ statistically significant association for women who had back pain, sometimes (*p <.005*).

^4^ statistically significant association for women who had back pain, often (*p <.005*).

**Table 4:** Back pain status and rating of conventional health care providers by CAM user status (consulted with a CAM practitioner or not)

	**Did not have back pain**		**Had back pain - rarely**		**Had back pain - sometimes**		**Had back pain - often**	
**Level of Satisfaction (1 = excellent, … , 5 = poor)**	**CAM non-user**	**CAM user**	**CAM non-user**	**CAM user**	**CAM non-user**	**CAM user**	**CAM non-user**	**CAM user**
	**(n = 1,682)**	**(n = 758)**	**(n = 1,419)**	**(n = 842)**	**(n = 1,950)**	**(n = 1,774)**	**(n = 970)**	**(n = 1,070)**
	**mean**	**mean**	**mean**	**mean**	**mean**	**mean**	**mean**	**mean**
**Access to a medical specialist if needed**	2.2	2.2	2.2	2.2	2.3	2.3	2.5	2.4
**Access to a female GP**	2.5	2.5	2.6	2.5	2.7	2.6	2.7	2.7
**Hours when a GP is available**^4^	2.7	2.8	2.8	2.8	2.9	2.9	2.8	3.0
**Number of GPs you have to choose from**	2.7	2.7	2.8	2.8	2.9	2.8	2.9	2.9
**Ease of seeing GP of your choice**^4^	2.7	2.9	2.8	2.9	2.9	2.9	2.8	3.0
**How long you wait to get a GP appointment**^4^	2.9	3.0	3.0	3.0	3.0	3.0	3.0	3.2
**The outcomes of your medical care (how much you are helped)**	2.3	2.4	2.4	2.5	2.4	2.4	2.5	2.6

^1^ Statistically significant association for women who did not have back pain (*p <.005*).

^2^ Statistically significant association for women who had back pain, rarely (*p <.005*).

^3^ Statistically significant association for women who had back pain, sometimes (*p <.005*).

^4^ Statistically significant association for women who had back pain, often (*p <.005*).

As shown in Table 4 there were significant associations between the rating of conventional care providers/provision and back pain status, by use of CAM (i.e. consultation with a CAM practitioner). Specifically, CAM users were significantly less satisfied with the hours when a GP is available (p <0.005), the ease of seeing a GP of choice (p <0.005), and the length of time they had to wait to get a GP appointment (p <0.005).

### Relationship between CAM use and health status

The association between CAM user status and the SF-36 dimensions of health status are presented for each back pain category separately in Table 5. For those women who experienced back pain, only their general health, physical functioning and vitality dimensions were significantly associated with CAM use (p <0.005). Specifically, women who experienced any back pain and consulted with a CAM practitioner had significantly better general health than women with back pain who did not consult a CAM practitioner. If we break down the back pain categories, those women who experienced back pain sometimes or often and consulted with a CAM practitioner had significantly better physical functioning than women who did not consult with a CAM practitioner. Women who experienced back pain often and consulted with a CAM practitioner had significantly better levels of vitality than women who did not consult with a CAM practitioner.

**Table 5:** Back pain status and SF-36 dimensions of health status by CAM user status (consulted with a CAM practitioner or not)

**SF-36 Dimensions of health status**	**Did not have back pain**		**Had back pain - rarely**		**Had back pain - sometimes**		**Had back pain - often**	
	**CAM non-user**	**CAM user**	**CAM non-user**	**CAM user**	**CAM non-user**	**CAM user**	**CAM non-user**	**CAM user**
	**(n = 1,682)**	**(n = 758)**	**(n = 1,419)**	**(n = 842)**	**(n = 1,950)**	**(n = 1,774)**	**(n = 970)**	**(n = 1,070)**
	**mean**	**mean**	**mean**	**mean**	**mean**	**mean**	**mean**	**mean**
**General health**^2 3 4^	78.7	78.8	75.2	78.8	70.4	72.9	54.5	58.2
**Physical functioning**^3 4^	87.2	87.3	85.1	86.7	80.1	82.2	60.2	66.0
**Bodily pain**^1^	82.1	77.8	77.0	76.4	67.2	66.1	43.3	44.9
**Role physical**^1^	87.5	83.5	84.5	82.9	76.3	76.8	47.8	51.4
**Role emotional**	89.8	87.5	86.8	85.9	82.9	84.8	68.6	71.2
**Mental health**^1^	81.2	78.9	78.5	79.3	75.2	76.3	66.1	68.1
**Social functioning**	89.3	87.2	87.8	87.5	84.2	84.3	68.2	70.9
**Vitality**^1 4^	69.1	66.6	65.1	65.9	59.2	60.1	43.9	46.6

^1^ Statistically significant association for women who did not have back pain (*p <.005*).

^2^ Statistically significant association for women who had back pain, rarely (*p <.005*).

^3^ Statistically significant association for women who had back pain, sometimes (*p <.005*).

^4^ Statistically significant association for women who had back pain, often (*p <.005*).

## Discussion

This nationally representative cross-sectional study is the largest exploration of treatment utilisation for Australian women with back pain to date. As a baseline, the study reveals the burden of back pain for mid-aged women in Australia with 77% (n = 8063) experiencing some form of back pain and 20% (n = 2044) often. The prevalence of back pain, with the associated consequences for mobility and general physical and mental health, strengthen the already well-documented identification of back pain as a major public health problem in Australia.

The study shows broadly that mid age women who consulted with a CAM practitioner have better general health, physical functioning and vitality. This finding supports those identified by Foltz et al [17] whose results indicate that CAM users with chronic back pain were healthier and more active than those who did not use CAM. While these women may have had better health status to begin with, they may also have been helped by CAM use providing better management of their back pain. Our analysis also reveals that those women who experienced back pain ‘often’ were less satisfied with access to general practitioners, perhaps pointing to a motivation for consulting with a CAM practitioner [17] (though usually only in concurrence with conventional care).

The use of CAM amongst this cohort of mid age women appears to be supplementary rather than exclusive. Most women consulted a conventional care provider but many also consulted with a CAM practitioner. The more frequent the back pain, the more likely they were to consult with a CAM practitioner as well as conventional care provider. This use of multiple practitioners reinforces findings from previous studies which have shown the use of conventional care along with CAM for the management of back pain [5,7,27]. The women in our study did not forgo their conventional care providers, but did concurrently use CAM practitioners. Importantly only 1.7% of women with back pain consulted with a CAM practitioner alone. As a conventional practitioner was almost always consulted by these back pain sufferers, any concerns regarding risk and CAM may be overstated, providing there is communication between patient and GP about CAM use. The increasing prevalence of CAM use, and the efficacy benefits – both evidenced and perceived – in line with the finding that the overwhelming majority of women who sought help for back pain utilised CAMs concurrently to conventional care, underlines the importance of mutual disclosure and discussion between patients and general practitioners [3].

Given that GPs are generally the first provider consulted for back pain care, the findings provide a timely reminder of the value of open communication with patients about their CAM use, in order to ensure agreement about therapeutic plans [13]. Better communication is needed between patients, conventional providers and CAM practitioners to ensure the creation and maintenance of ‘best’ treatment plans for back pain sufferers. Our findings point to the relevance of future research aimed at understanding and exploring the nature of treatment utilisation, particularly whether treatments are self-administered, provided by practitioners, or self-administered with the aid of a practitioner. Moreover, they point to the importance research focused on enhancing our understandings of patient motivations and treatment utilisation for back pain and the determinants of care-seeking [28] as well as patient and practitioner experiences of communication about the utilisation of a range of therapies.

We acknowledge the limitations of our study, firstly in the potential effects of recall bias following the use of self reporting of health and treatment utilisation by the participants. Additionally, back pain status was defined by the self-reporting of a single question. This lack of confirmatory diagnosis could potentially bias the findings, however, existing research has evidenced the validity, and comparability to medical record assessments, of a questionnaire-based measure of comorbidity [29]. A final limitation lies in our inability to ascertain whether the self-reported use of CAM and conventional healthcare was for back pain or some other reason(s). This limitation though is offset by the analyses of such a large, nationally representative sample of mid-age women. Moreover, our findings strongly suggest the use of individual CAM modalities for back pain care. For all CAM provider modalities consultations increased as the frequency of back pain increased, with the most utilised CAM practitioner groups (massage therapy and chiropractic) acknowledged as popular CAM therapies for back pain [21].

## Conclusions

For mid-aged women with back pain in Australia, a range of conventional and CAM treatments are utilised. Consultation with CAM practitioners or self-prescribed CAM was predominantly in addition to, rather than a replacement for, conventional care. The results reinforce the need for effective and ongoing communication between patients, conventional and CAM practitioners to ensure the creation and maintenance of treatment plans for back pain sufferers.

## Endnotes

^a^Ethics clearance number: 2004000224

^b^Ethics Clearance number: H0760795

^c^For the purposes of this study, and this paper, we define CAM according to practitioner groups not traditionally associated with the conventional medical profession or curriculum.

^d^Participants were asked to self assess their back pain in the last 12 months according to the response options of: “Never”, “Rarely”, “Sometimes” or “Often”.

## Abbreviations

CAM: Complementary and alternative medicine; GP: General practitioner; ALSWH: Australian Longitudinal Study on Women’s Health.

## Competing interests

The authors declare that they have no competing interests.

## Author’s contributions

AB, DS and JA conceived of the study, and supervised the data collection. AB, EK, DS, JA and KR participated in the design of the study. DS led the statistical analysis. All authors were involved in and contributed to the data analysis. All authors participated in drafting and revising the manuscript, and all authors approved the final manuscript.

## Pre-publication history

The pre-publication history for this paper can be accessed here:

http://www.biomedcentral.com/1472-6882/12/98/prepub
